# Genetic Differentiation, Structure, and a Transition Zone among Populations of the Pitcher Plant Moth *Exyra semicrocea*: Implications for Conservation

**DOI:** 10.1371/journal.pone.0022658

**Published:** 2011-07-28

**Authors:** Jessica D. Stephens, Scott R. Santos, Debbie R. Folkerts

**Affiliations:** 1 Department of Biological Sciences, Auburn University, Auburn, Alabama, United States of America; 2 Molette Biology Laboratory for Environmental and Climate Change Studies, Auburn University, Auburn, Alabama, United States of America; 3 Cell and Molecular Biosciences Peak Program, Auburn University, Auburn, Alabama, United States of America; Biodiversity Insitute of Ontario - University of Guelph, Canada

## Abstract

Pitcher plant bogs, or carnivorous plant wetlands, have experienced extensive habitat loss and fragmentation throughout the southeastern United States Coastal Plain, resulting in an estimated reduction to <3% of their former range. This situation has lead to increased management attention of these habitats and their carnivorous plant species. However, conservation priorities focus primarily on the plants since little information currently exists on other community members, such as their endemic arthropod biota. Here, we investigated the population structure of one of these, the obligate pitcher plant moth *Exyra semicrocea* (Lepidoptera: Noctuidae), using mitochondrial cytochrome *c* oxidase subunit I (COI) gene sequences. Examination of 221 individuals from 11 populations across eight southeastern US states identified 51 unique haplotypes. These haplotypes belonged to one of two divergent (∼1.9–3.0%) lineages separated by the Mississippi alluvial plain. Populations of the West Gulf Coastal Plain exhibited significant genetic structure, contrasting with similarly distanced populations east of the Mississippi alluvial plain. In the eastern portion of the Coastal Plain, an apparent transition zone exists between two regionally distinct population groups, with a well-established genetic discontinuity for other organisms coinciding with this zone. The structure of *E. semicrocea* appears to have been influenced by patchy pitcher plant bog habitats in the West Gulf Coastal Plain as well as impacts of Pleistocene interglacials on the Apalachicola-Chattahoochee-Flint River Basin. These findings, along with potential extirpation of *E. semicrocea* at four visited, but isolated, sites highlight the need to consider other endemic or associated community members when managing and restoring pitcher plant bog habitats.

## Introduction

Currently, global biodiversity losses are occurring at an alarming rate, exceeding anything in the geological past [1]. These losses have further implications as they can exacerbate collapses of ecosystem functions. The most recognized and potentially well-documented examples of biodiversity and ecosystem loss have been in the tropics, home to half of the world's species [1]. However, less well known is the fact that temperate zone ecosystems including marine [2], freshwater [3], and terrestrial [4] environments are being adversely impacted at an even faster rate. Given that the majority of the world's affluent human populations have historically resided in the temperate region [5], [6], it is not surprising that many of its ecosystems have experienced anthropogenic driven environmental degradation, extensive species declines and habitat loss [7]–[9]. Such negative anthropogenic influences are well documented to have occurred in the United States, which has ∼38 threatened, 58 endangered, and more than 30 critically endangered ecosystems [10].

Among the most critically endangered in the United States is the longleaf pine ecosystem, which historically occupied most of the southeastern Coastal Plain [10]. Estimates suggest that there has been a >98% decline of pre-European settlement longleaf pine (*Pinus palustris*) forests in this area [11]. Encompassed within the longleaf pine ecosystem are a myriad of endangered habitats including carnivorous plant wetlands, commonly referred to as *pitcher plant bogs*. These bogs are typically found in wet pine flatwoods or seepage slopes and are characterized by wet, sandy and low nutrient soils [12]. In these habitats, many endemic plants have evolved a carnivorous lifestyle, obtaining nutrients through the capture and digestion of animal prey. Similar to the overall ecosystem, current estimates suggest that the pitcher plant habitat now occupies <3% of its former range largely due to restriction of natural fires, urbanization, forestry and agriculture [12]. Of the more than 29 carnivorous plant species representing five genera [13], at least five species are federally endangered or threatened [14] and all species in the genus *Sarracenia* (pitcher plants) are listed in The Convention on International Trade in Endangered Species (CITES, www.cites.org).

Many studies on carnivorous plants have focused on the investment in and evolution of carnivory [15]–[17] that was pioneered in part by Charles Darwin's fascination with the subject [18]. Other areas of interest include the genetics and population structure of these plant species since such information has implications for conservation of these unique organisms. For example, varying levels of genetic diversity have been reported across *Sarracenia* sp. and populations [19]–[21]. Additionally, recent research examining *Sarracenia alata* (the winged pitcher plant), which ranges from eastern Texas to western Alabama, identified a phylogeographic break and high genetic divergence centered on the Mississippi River alluvial plain, with additional population structure on either side of the break [22]. Such results highlight the potential uniqueness of individual pitcher plant populations that should be considered when developing management plans for these habitats.

While research and conservation efforts have primarily focused on the carnivorous plants, less attention has been given to other constituents of pitcher plant bogs. This is unfortunate because these bogs support a complex and intimately associated biotic community. For example, a number of arthropods have evolved characteristics for inhabiting *Sarracenia* sp. [12], including >17 species of endemic mites, flies and moths [13], [23], [24]. Of these, the herbaceous moth *Exyra semicrocea* (Lepidoptera: Noctuidae) ranges across the United States southeastern Coastal Plain from eastern Texas to southern Virginia [25], [26] and is of conservation concern due to its obligate relationship with *Sarracenia* sp. like *S. alata* [25], [27]. Specifically, *E. semicrocea* is oligophagous, feeding exclusively on *Sarracenia* sp., and its life cycle occurs entirely inside pitcher plant leaves, from oviposition through larval development and mating. Because of this well documented relationship, we believe that the *E. semicrocea/Sarracenia* complex represents an ideal system for examining ecological and biogeographical questions pertaining to pitcher plant bogs and their endemic biota. Here, we examined populations of *E. semicrocea* across the southeastern Coastal Plain to address the hypothesis that the genetic structure of this moth is consistent with the major finding of Koopman and Carstens [22] for *S. alata*, namely that a strong genetic break occurs across the geographic region occupied by the Mississippi River alluvial plain.

## Methods

### Ethics Statement

This study was conducted in accordance with all United States laws and internationally accepted protocols with relevant collecting permits from The Nature Conservancy (for sites Centre Bog, Eller Seep, Green Swamp, Reed Branch), National Estuarine Research Reserve Sites (covered under Award/Permit #NA10NOS4200038; for sites Apalachicola, Grand Bay, Weeks Bay), Big Thicket National Preserve Site (Permit # BITH-2010-SCI-0006; for site Big Thicket), and Okefenokee National Wildlife Refuge (Permit # 41590-10-033; for site Okefenokee).

### Sample techniques, DNA extraction and polymerase chain reaction (PCR)

**Figure 1:**
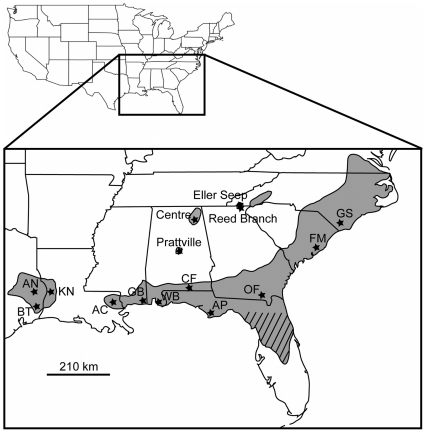
Map depicting the range of pitcher plant bogs and *Exyra semicrocea* across the southeastern United States Coastal Plain. Shaded regions represent historical range while hash marks indicate areas that no longer possess contemporary pitcher plant bog habitats. Sampling locations of *Exyra semicrocea* populations examined in this study are indicated by stars. The three localities west of the Mississippi River are Angelina National Forest (AN), Big Thicket Nature Preserve (BT), and Kisatchie National Forest (KN). Localities sampled across the eastern portion of the range are Abita Creek Flatwoods Preserve (AC), Grand Bay National Estuarine Research Reserve Site/National Wildlife Refuge (GB), Weeks Bay National Estuarine Research Reserve Site (WB), Conecuh National Forest (CF), Apalachicola National Estuarine Research Reserve Site/National Forest (AP), Okefenokee National Wildlife Preserve (OF), Francis Marion National Forest (FM), and Green Swamp Preserve (GS). Bogs at Centre, Eller Seep, Prattville, and Reed Branch were visited, but no *E. semicrocea* were located.

Specimens of *E. semicrocea* were collected across eight southeastern Coastal Plain states between May and August 2010 (Figure 1). *Exyra semicrocea* was not found at four of the visited sites (Centre, AL; Prattville, AL; Reed Branch, GA and Eller Seep, NC); thus, samples were acquired from 11 localities. At each site, attempts were made to collect a minimum of 24 *E. semicrocea* individuals, however, the actual number acquired per population ranged from 3–24 (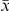 = 21) specimens. Individuals were preserved in 95% ethanol in the field and DNA extraction from each *E. semicrocea* followed the methods described by Coffroth et al. [28]. The resulting DNA extractions served as templates for amplifying a ∼680 bp fragment of the mitochondrial (mtDNA) cytochrome *c* oxidase subunit I (COI) gene. The primer pair for subsequent polymerase chain reaction (PCR) was LCO1490 (5′-GGTCAACAAATCATAAAGATATTGG-3′) and HCO2198 (5′-TAAACfTTCAGGGTGACCAAAAAATCA-3′) [29]. The COI region was chosen because it can reveal both historical and current genetic patterns [30] and has been shown to be informative in other Lepidoptera species [31], [32]. Each PCR was conducted in 25 µl volumes containing ∼10–30 ng of template DNA, 10 mM Tris-HCl (pH 8.3), 50 mM KCl, 0.001% gelatin, 2.0 mM MgCl_2_, 200 mM dNTPs, 1 U *Taq* DNA polymerase and 0.4 mM of each primer. PCR was conducted with a PTC-100 Thermal Cycler (MJ Research) using the following cycling profile: initial denaturing step of 94°C for 5 min, 15 cycles of 94°C for 45 s, 40°C for 45 s, 72°C for 60 s; 25 cycles of 94°C for 45 s, 55°C for 45 s, 72°C for 60 s, and a final extension of 72°C for 5 min. Five µl aliquots of PCR product from each reaction were electrophoresed in 1% agarose gels, stained using ethidium bromide, and viewed using shortwave (265 nm) UV to confirm PCR success. Prior to sequencing, amplification products were purified with Montage™ PCR Filter Units (Millipore) according to the manufacturer's specifications. Sequencing was conducting in both directions using Big-Dye Terminators v.3.1 and analyzed on a PRISM 3100 (Applied Biosystems, USA) at the Auburn University Genomics and Sequencing Laboratory or the University of Washington Genomics Unit. Forward and reverse sequence chromatograms were assembled and any ambiguities corrected using Sequencher v4.8 (Gene Codes Corporation, USA). All finished sequences were aligned manually using SE-AL version v2.0a11 (available at http://tree.bio.ed.ac.uk/software/seal/).

### Data analysis

#### Genetic diversity, population structure and migration

Estimates of population genetic diversity were obtained using haplotype (*h*) and nucleotide (π) diversities calculated by Nei's [33] method in the program DnaSP v5.10.01 [34]. Population structure was estimated using two approaches. First, the S_nn_ or ‘nearest-neighbour’ statistic [35], [36] was calculated as pairwise comparisons among sites, with 1,000 permutations to assess statistical significance, in DnaSP. Next, pairwise Φ*_ST_* statistics based on haplotype frequency and molecular diverfogence were generated using Arlequin v3.1 [37], [38]. Notably, since *E. semicrocea* were collected from four species of *Sarracenia* (i.e., *S. alata*, *S. flava*, *S. leucophylla*, *S. minor*), S_nn_ and pairwise Φ*_ST_* statistics were also conducted to test whether *E. semicrocea* exhibited genetic structure between host plant species. A lack of significant structuring was detected among these comparisons (data not shown), thus the results presented here focus specifically on genetic structure between geographic sites. To quantify the spatial distribution of genetic variation in *E. semicrocea*, analyses of molecular variance (AMOVAs) [39] were conducted with Arlequin. Estimates of the relative contribution of molecular variance were assessed at three hierarchical levels using Φ-statistics: (i) between groups (Φ*_CT_*, designated by the resulting population structure from pairwise Φ*_ST_* and S_nn_ statistics); (ii) among populations within groups (Φ*_SC_*); and (iii) within populations (Φ*_ST_*). The significance of both the pairwise Φ*_ST_* statistics and AMOVAs were assessed with 10,000 permutations. All of the above tests, when applicable, were conducted under Tamura and Nei's [40] model of nucleotide evolution with rate variation among sites [TN+Γ] as selected by the Akaike Information Criterion (AIC) in ModelTest v3.6 [41]. Lastly, Mantel [42] and 3-way (partial) Mantel [43] tests were conducted with the Vegan package [44] in the R v2.6.2 statistical software environment [45] with 10,000 permutations. A standard Mantel test was utilized to assess whether genetic and geographical distances are correlated. To determine if a potential transition zone among eastern populations influences the relationship between genetic and geographical distances, partial Mantel tests were conducted with an additional binary matrix defining populations as belonging to one of two groups (see Results).

To discriminate between the relative effects of ongoing migration (M = 2N*_ef_m*) versus recent divergence between pairs of *E. semicrocea* populations, a Markov Chain Monte Carlo (MCMC) based analytical method was utilized as implemented in mdiv [46] (available at http://cbsuapps.tc.cornell.edu/). Analyses were conducted under the finite-site mutation model, which incorporates the possibility of multiple mutations per site, differences in nucleotide frequencies and the presence of transition/transversion bias. Three independent runs were conducted with the following conditions: *M_max_* = 50, *T_max_* = 10, length of Markov chain = 2×10^6^ cycles, burn-in time = 5×10^5^ cycles. Different random seeds were used in each run to check for consistency in the estimates.

#### Haplotype network and demographic analyses

Networks were generated to depict relationships among *E. semicrocea* haplotypes, based on the cladogram estimation algorithm of Templeton et al. [47], with tcs v1.21 [48]. The networks were constructed under the default setting (i.e., 95% parsimonious plausible branch connections). Reticulations in the networks, implying ambiguous connections between haplotypes, were resolved according to the suggestions of Crandall et al. [49]. Two approaches employing Fu's *F*_S_ [50] and Tajima's *D* [51] were used to determine if patterns of genetic variation observed in the COI sequences were consistent with predictions under a neutrality model. While these tests are typically employed to detect selection [50], [51], they can also prove informative regarding the demographic history of a population [52]. Both neutrality tests were conducted in Arlequin and significance (*P*<0.05) assessed by 10,000 permutations.

## Results

### Genetic diversity of *Exyra semicrocea*

A total of 221 *Exyra semicrocea* were sampled from 11 localities across a range of ∼1,700 km. Overall, 51 (8.1%) nucleotide positions across the analyzed 630 bp COI fragment were variable and no stop codons were encountered. Nucleotide differences at the majority of these sites (50) represented ‘silent’ substitutions (i.e., would not change the encoded amino acid), with the remaining one being a non-synonymous substitution to an amino acid with similar biochemical properties (data not shown). These patterns suggest that all sequences were derived from mitochondrial copies of COI rather than nuclear copies of mitochondrial derived genes (numts) [53], [54]. From the 221 total individuals sampled, 54 unique haplotypes were identified with 34 occurring as singletons and the remaining 23 being identified more then once (See Table S1). Sequences were deposited in GenBank under accession numbers HQ646110–HQ646163.

**Figure 2:**
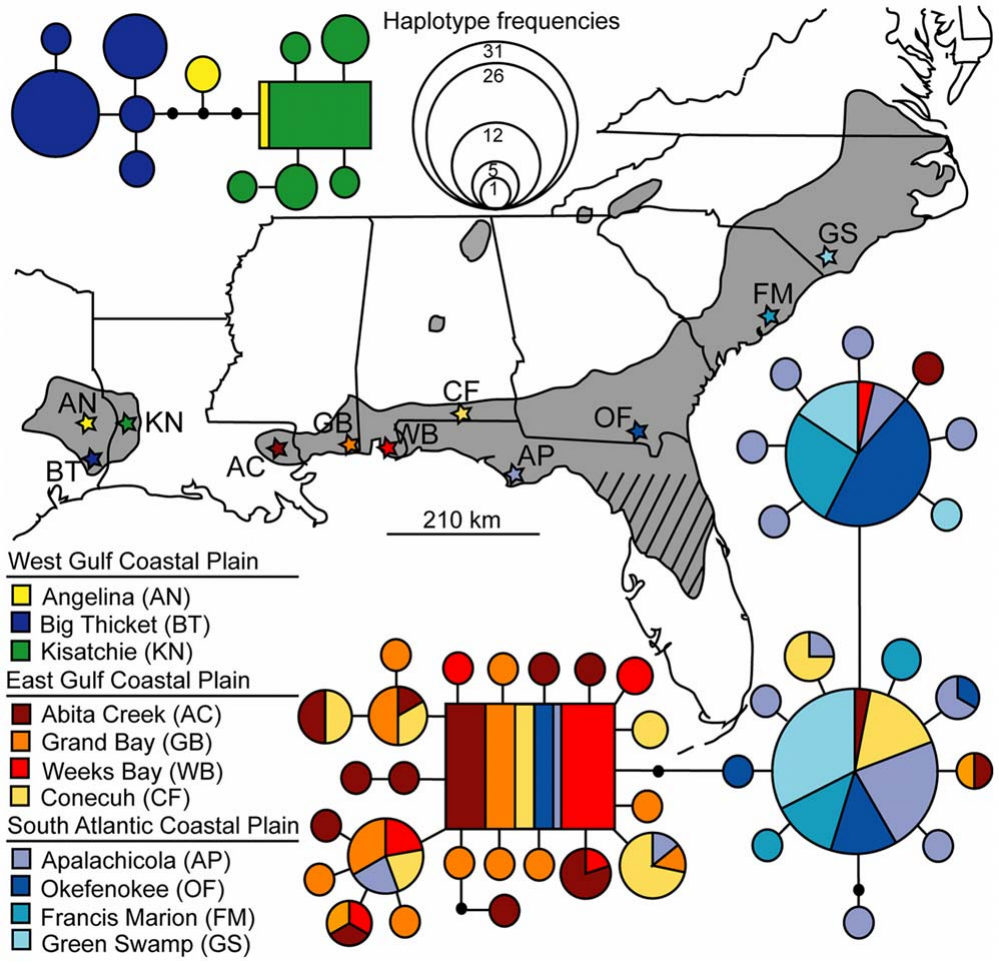
Map and corresponding haplotype networks for the western and eastern lineages of *Exyra semicrocea* across the southeastern United States Coastal Plain. Each circle in a network represents a unique mitochondrial DNA (mtDNA) cytochrome *c* oxidase subunit I (COI) haplotype, with circle size corresponding to haplotype frequency. Rectangles represent the inferred ancestral haplotype or the haplotype with the highest outgroup probability according to the tcs analyses. Each branch, in spite of length, represents one mutational difference between haplotypes, with black circles indicating unsampled (i.e., missing) haplotypes. Colors represent localities designated by matching stars, which correspond to sampling sites specified in Figure 1.

Haplotype frequencies within populations varied from 2–15, with haplotype (*h*) and nucleotide (π) diversities ranging between 0.51–0.94 and 0.00097–0.00434, respectively (Table 1). Genetic distances between any two haplotypes ranged from 1 (0.2%) to 19 (3.0%) variable sites, with sequence divergence across the Mississippi River alluvial plain being between 1.9–3.0% (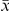 = 2.5%). Arthropod COI mutation rates have been estimated at 2.3% sequence divergence per million years [55], and more specifically, between 0.78–1.02% per million years for the family Papilionidae (Lepidoptera) [56]. Use of these rates yield an estimate that populations separated by the Mississippi River alluvial plain diverged from each other between 1.0–3.2 mya. Furthermore, none of the identified haplotypes were shared between populations across the Mississippi River alluvial plain (Figure 2). Thus, all subsequent population genetic analyses treated the western and eastern populations of *E. semicrocea* separately.

**Table 1:** Genetic diversity and neutrality tests for *Exyra semicrocea* populations across the southeastern United States Coastal Plain.

		Genetic diversity				Neutrality tests	
Geographic region	Population	*n*	*nh*	π	*h*	Tajima's *D*	Fu's *F*_S_
West Mississippi R.	AN	3	2	0.00317	0.66667	0.000	1.609
	BT	24	5	0.00189	0.67754	0.309	−0.432
	KN	24	6	0.00149	0.70290	−0.867	−2.162*
Total	3	51	13	0.00520	0.86510	0.158	−1.921
East Mississippi R.	AC	24	14	0.00411	0.89855	−1.548*	−7.981*
	GB	23	14	0.00305	0.91700	−2.002*	−10.786*
	WB	24	7	0.00167	0.55797	−1.957*	−3.265*
	CF	24	8	0.00434	0.89130	0.437	−7.981*
	AP	24	15	0.00400	0.91304	−1.465	−9.957*
	OF	21	5	0.00230	0.64286	0.125	−0.102
	FM	15	4	0.00160	0.71429	0.280	−0.064
	GS	15	3	0.00097	0.51429	−0.024	−0.064
Total	8	170	44	0.00457	0.89795	−1.632*	−26.374*

*n*, number of sampled individuals.

*nh*, number of recovered haplotypes.

π, nucleotide diversity.

*h*, haplotype diversity.

* *P*<0.05.

### Genetic differentiation, structure, and migration rates

Pairwise Φ*_ST_* and S_nn_ statistics revealed significant genetic differentiation in 46 of 62 pairwise population comparisons of *E. semicrocea* (Tables 2 and 3). For example, all three populations west of the Mississippi River alluvial plain (KN, BT and AN: Fig. 1) were significantly differentiated from one another for both estimates, with comparisons between BT and KN (∼150 km) and BT and AN (∼75 km) having S_nn_ values of 1.0 (Table 2). This implies a complete lack of haplotype exchange between these populations. Estimates of migration (*M*) via mdiv further support this, suggesting no female (since mtDNA is maternally inherited) migrants (*M* = 0) had been exchanged between the BT and KN populations in the recent past (data not shown). An analysis of molecular variance (AMOVA) found a significant proportion of genetic variation occurring between populations (i.e., BT vs. AN/KN; ∼55%; Φ*_CT_* = 0.552; *P*<0.05). Additionally, estimates for among populations within groups and within populations were significant (*P*<0.01) and accounted for ∼24% and ∼21% of the total observed genetic variation, respectively (Table 4).

**Table 2:** Measures of genetic differentiation for western populations of *Exyra semicrocea* in Texas and western Louisiana as estimated by S_nn_ (upper right triangle) and pairwise Φ*_ST_* (lower triangle) statistics.

Populations	KN	BT	AN
KN	–	1.000***	0.920*
BT	0.800*	–	1.000***
AN	0.548*	0.694*	–

*P<0.05;

***P<0.001.

**Table 3:** Measures of genetic differentiation for eastern populations of *Exyra semicrocea* as estimated by S_nn_ (upper right triangle) and pairwise Φ*_ST_* (lower triangle) statistics.

Populations	AC	GB	WB	CF	AP	OF	FM	GS
AC	–	0.565	0.555	0.663**	0.795***	0.795***	0.897***	0.900***
GB	0.012	–	0.527	0.643**	0.789***	0.830***	0.974***	0.974***
WB	0.012	0.008	–	0.796***	0.797***	0.830***	0.949***	0.944***
CF	0.052	0.100*	0.137*	–	0.661**	0.784***	0.858***	0.817***
AP	0.380*	0.470*	0.524*	0.242*	–	0.548	0.617*	0.543
OF	0.470*	0.575*	0.647*	0.344*	0.009	–	0.572	0.591*
FM	0.558*	0.661*	0.751*	0.434*	0.045	0.047	–	0.562*
GS	0.564*	0.674*	0.773*	0.430*	0.017	0.065	0.005	–

*P<0.05;

**P<0.01;

***P<0.001.

**Table 4:** Analyses of molecular variance (AMOVAs) for *Exyra semicrocea* populations across the southeastern United States Coastal Plain.

Geographic region	Source of variation	df	Sum of squares	Variance component	% var.	Φ statistic
West Mississippi R.	Between groups	1	51.275	1.442	55.21	Φ_CT_ = 0.552*
	Among pops within groups	1	3.835	0.615	23.55	Φ_SC_ = 0.526**
	Within populations	48	26.619	0.555	21.24	Φ_ST_ = 0.788***
		50	81.728	2.611		
East Mississippi R.	Among groups	2	88.282	0.826	46.90	Φ_CT_ = 0.469**
	Among pops within groups	3	5.927	0.013	0.75	Φ_SC_ = 0.014***
	Within populations	162	149.409	0.922	52.35	Φ_ST_ = 0.477***
		169	243.663	1.762		

* = *P*<0.05;

** = *P*<0.01;

*** = *P*<0.001.

Comparisons among the eastern populations found a lack of genetic structure among AC, GB, and WB for both the pairwise Φ*_ST_* and S_nn_ statistics (Table 3). For AP, OF, FM, and GS, weak but significant structure was detected depending on the statistical estimate (Table 3). In contrast, both statistics identified CF as being distinct from nearly all other sites with the exception of AC (Table 3). Therefore, subsequent genetic analyses were conducted on the eastern population groups of AC/GB/WB, AP/OF/FM/GS and CF. In this context, a significant proportion of variation was partitioned within populations (∼52%; Φ*_ST_* = 0.523; *P*<0.001) and among groups (∼47%; Φ*_CT_* = 0.469; *P*<0.05), with a small (i.e., 0.75%; Φ*_SC_* = 0.014) but significant (*P*<0.001) component among populations within groups (Table 4). Furthermore, a Mantel test identified an overall positive (*r* = 0.542) and significant (*P* = 0.013) correlation between genetic and geographical distances. Because of the distinct geographic clustering observed among specific eastern populations, a 3-way Mantel test was conducted in order to evaluate the relative contributions of contemporary isolation-by-distance versus historical isolation to the overall pattern. For this analysis, a third binary (i.e., 1 = both populations in same group, 0 = different group) matrix was created and the 3-way Mantel test detected a significant correlation (*r* = 0.899, *P* = 0.024) between genetic distance and this binary (i.e., the matrix defining the two groups with 3–4 populations, CF was excluded) but no significance correlation (*r* = 0.122, *P* = 0.218) between the geographic and genetic distance matrices. These results suggest that historical isolation, rather than geographic distances, is most likely responsible for segregating the eastern populations into two primary groups. Taken together, these results indicate a transition, approximately located in the western Florida panhandle/southern Alabama between WB and AP (Figure 2), exists between the two groups of eastern *E. semicrocea*.

**Figure 3:**
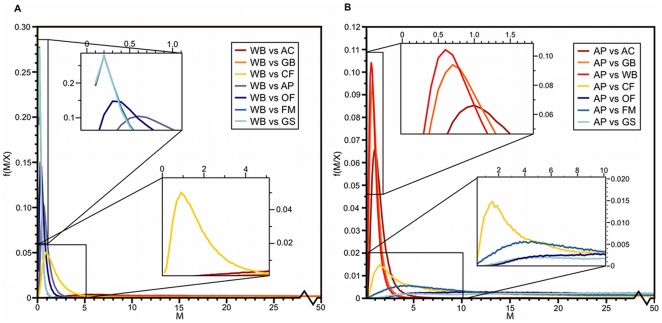
Posterior probability distributions of ongoing migration (*M*) for *Exyra semicrocea* populations in the southeastern United States Coastal Plain. Presented distributions are an average of three independent runs utilizing identical starting conditions but different random seeds from the program mdiv (Nielsen and Wakeley 2001). Migration (*M*) estimates for pairwise comparisons between A) Weeks Bay (WB) and B) Apalachicola (AP) relative to all other eastern *E. semicrocea* populations. Colors correspond to those utilized in locality designations of Figure 2.

Due to the occurrence of CF within the proposed transition zone (Figure 2), pairwise estimates of migration (*M*) between all eastern populations were conducted relative to both AP and WB (i.e., the closest populations to CF). Population comparisons on either side of the potential transition zone (i.e., within groups) revealed plateaus in the distribution of posterior probabilities of *M* (Figure 3), suggestive of high levels of intra-regional migration and consistent with the lack of genetic structure implied by the S_nn_ and Φ*_ST_* statistics for these populations (see above). Conversely, clear peaks in the distribution of posterior probabilities for *M* were identified in population comparisons across the proposed transition zone (i.e., between groups; Figure 3). For example, population comparisons of WB relative to GS, FM, OF, and AP (i.e., across groups) produced estimates for *M* of 0.2, 0.2, 0.3, 0.5, respectively, with plateaus for AC and GB (i.e., within groups; Figure 3A). These results were inversed for population comparisons of AP (Figure 3B), which is located on the opposite side of the proposed transition zone (Figure 2). In both cases, CF remained an outlier with an estimated *M* approximately double that of population comparisons across the proposed transition zone (i.e., between groups; Figure 3). Again, these estimates of *M* imply a distinct shift in the level of migration in relation to the proposed transition zone, a pattern consistent with the outcome of the 3-way Mantel test.

### Demography of *Exyra semicrocea*

Populations west of the Mississippi River alluvial plain had both positive and negative values for Tajima's *D* and Fu's *F*_S_, with KN being the only population with a significant negative *F*_S_ value (Table 1). In contrast, five of eight populations east of the Mississippi River possessed significant negative *F*_S_ values, with three (i.e., AC, GB, WB) also significant for Tajima's *D* (Table 1). Significant negative values indicate an excess of rare polymorphisms within each of these populations and implied either positive selection or recent population expansion [51], [52]. Due to the near total absence of non-synonymous substitutions in the analyzed COI fragment (see above), it is surmised that recent population expansion is the most likely explanation for these values. Taken together, populations of *E. semicrocea* in the eastern portion of its range as a relative whole are undergoing expansion compared to those west of the Mississippi River alluvial plain (Table 1).

## Discussion

### West Gulf Coastal Plain and Mississippi River alluvial plain

Populations of *Exyra semicrocea* in the West Gulf Coastal Plain (WGCP, USGS designation) possess appreciable divergence from, and a clear break between, eastern populations across the Mississippi River alluvial plain. The identification of the Mississippi River alluvial plain as a significant barrier has been documented for a number of species [57], [58], including other members of the longleaf ecosystem, such as *Pinus palustris* (longleaf pine), *Dichromona colorata* (white topped sedge), *Drosera capillaris* (pink sundew), *Rhexia alifanus* (a meadow beauty), and *Utricularia* sp. (bladderworts) [59]. Notably, an identical break is present in the host plant of western *E. semicrocea* in this region (i.e., *Sarracenia alata*) [22] and results from an ∼200 km wide swath of unsuitable soils and alluvial swamps replacing bog habitat in this region [60]. Given that *S. alata* minimally overlaps with other *Sarracenia* sp. throughout its range [61] and is the only host plant for populations of *E. semicrocea* in Texas and Louisiana, it is not surprising that this barrier has influenced a similar pattern in both species. Interestingly, the genetic structure of microbial communities found within the pitchers of *S. alata* also mirrors that of the plant [62] and, by extension, that of *E. semicrocea* (this study). Taken together, these patterns highlight the biological uniqueness and intimate associations among constituents of pitcher plant bog habitats in the WGCP.

In the west, the significant genetic structure among geographically close populations of *E. semicrocea* contrasts with an absence of genetic structure among similarly (or more widely) distanced populations in the east (Figure 2, see above). One explanation for this may be a lack of historical connectivity between patches of bogs in the west. While little is known concerning the history of pitcher plant bogs in this region, it is estimated that bogs of the WGCP may have occupied ∼4,000–8,000 ha prior to European settlement [63] and been distributed among sites in as many as 20 counties (e.g., 4 parishes in Louisiana, 16 counties in Texas) [60]. However, areas that currently harbor locally abundant pitcher plant bogs are just two parishes in Louisiana (which encompasses KN) and four counties in Texas (which encompasses AN and BT). Unfortunately while historical records of small and/or rare populations can provide useful information for establishing past distributions, they may also artificially extend “known” ranges due in part to the reporting of erroneous localities, producing a false picture of historical abundance in the process [64]. Such a situation has been reported for pitcher plant records in Texas [60] and if this is the case, pitcher plant bogs in the WGCP might have been as patchy and historically isolated as they are today. Along with this, it is well known that floristic composition differs between eastern and western portions of the Coastal Plain [65], [66], with these differences typically attributed to variations in soil [65] and levels of rainfall [67]. Additionally, many western bogs are supported by springs [67] rather then rainfall, the more common water source for their eastern counterparts. Such environmental differences may have contributed to habitat patchiness and the paucity of large, expansive bogs in the west relative to the east and are potential contributors to the local and significant population structure of *E. semicrocea* that likely extends to other obligate pitcher plant bog organisms as well.

Another explanation for the significant structure among western *E. semicrocea* populations is a possible difference in dispersal behavior resulting from genetic differentiation. Specifically, the COI sequence divergence between *E. semicrocea* from the WGCP and eastern Coastal Plain ranges from 1.9–3.0% and while sequence divergence values for Lepidoptera congeneric species pairs are generally greater than 3.0% [68], values for Lepidoptera species known to be morphologically different can be lower than 3.0% [69]. Thus, the level of difference seen here indicates a period of isolation between western and eastern *E. semicrocea* populations during which it is conceivable that behavioral differences may have evolved. If so, further investigations into potential morphological and behavioral differences between the eastern and western lineages of *E. semicrocea* are warranted since such information has important implications in future conservation efforts (see below).

### Eastern population structure and Apalachicola-Chattahoochee-Flint River (ACF) break

Populations of *E. semicrocea* east of the Mississippi River alluvial plain are genetically structured into three distinct groups. These groups are situated within the East Gulf Coastal Plain (EGCP, USGS designation, encompassing AC/GB/WB), the South Atlantic Coastal Plain (SACP, USGS designation, encompassing AP/OF/FM/GS), and Conecuh National Forest (CF) in the proposed transition zone between the groups (although it should be noted that CF is officially within the East Gulf Coastal Plain USGS designation, Figure 1). Notably, this transition zone includes the Apalachicola-Chattahoochee-Flint (ACF) River basin, a well-documented area for genetic discontinuity for a number of vertebrates [70], invertebrates [71], and plant [72] species. Various hypotheses on why the ACF is an area of genetic discontinuity have been proposed. One of these argues that the Apalachicola River and its resulting floodplain is one of the few systems completely bisecting the eastern Coastal Plain and therefore likely both a historical and contemporary physical barrier [73]. In addition, this system is responsible for draining the Appalachian Mountain massif, creating distinct soil differences on the resulting sides of the ACF [65]. Along with this, areas of the Coastal Plain were consistently inundated by the Gulf of Mexico during the late Pliocene and Pleistocene interglacial periods and it is thought that while these sea level increases did not completely cover the entire Coastal Plain, range fragmentations occurred due to coastal advances and the inability of endemic taxa to shift their ranges further north [73]. Under this scenario, refugia were likely to have been created both west and east of the ACF for these taxa during interglacial periods in the Pleistocene.

While a general pattern of discontinuity at the ACF basin is well established, it is not necessarily congruent across taxa. In this study, *E. semicrocea* is structured across the ACF, but with CF behaving as an outlier by possessing haplotypes commonly found both west and east of the ACF basin (Figure 2). In addition, migration estimates at CF undergo a distinct transition, suggesting the influence of a potential barrier in this area. However, the exact mechanism(s) contributing to these trends in *E. semicrocea* are difficult to identify since the region between the Mississippi and Apalachicola Rivers has a history of variable phylogeographic patterns across many species [72]. For example, studies of southeastern Coastal Plain fishes [70], [74] and other vertebrate taxa [70], [72] highlight some of the inconsistencies in the genetic structure of organisms from this region. Overall, this variability is likely a product of numerous interglacial events spanning the Pleistocene that resulted in refugia and divergence in isolation. Given this, future phylogeographic studies of additional taxa from this region, including *Sarracenia* species and its associated arthropods, may provide further insight toward resolving the unusual nature of this area.

With the exception of CF, the other EGCP populations (i.e., AC, GB, WB) of *E. semicrocea* exhibit demographic expansion but no genetic structure among them, even though the distances between populations are similar to those west of the Mississippi River (Figure 1). These differences are likely due to much more contiguous bog habitat, both historically and contemporarily, across the EGCP. Specifically, early writings imply a large expanse of bog, possibly stretching from Pensacola, Florida, west to Pascagoula, Mississippi (a span of 130 km), before the late 1800s [75], [76]. Support for this comes from habitat suitability assessments and soil surveys of the EGCP indicating pitcher plant bogs might have occupied 293,500 ha across the region in pre-Columbian times [65]. In contrast to the EGCP, populations of *E. semicrocea* in the SACP (i.e., AP, OF, FM, GS) exhibited in some cases weak, but significant, genetic structure (Table 3). One potential driver for this pattern might again be habitat patchiness. For example, sites such as OF and FM have minimal pitcher plant bog habitat due to large swaths of historic swampy areas. Furthermore, in the northern portions of the SACP (i.e., South and North Carolina), regionally specific “bog-like” habitats known as pocosins and Carolina Bays either exclude (i.e., pocosin) or create elliptical, irregular pitcher plant habitats (i.e., Carolina Bays) [65]. These alternative habitat types as well as the effects of glacial advances and retreats over time in this region may contributed to the generation of pitcher plant bogs with patchy distributions and initiated the process of differentiation among *E. semicrocea* populations in the SACP.

### Conservation Implications

As mentioned previously, pitcher plant bogs of the southeastern United States Coastal Plain possess the most diverse assemblages of carnivorous plants in the world [13] while also belonging to one of the most critically endangered ecosystems in North America [10]. Thus, it is imperative to further develop sound management practices for maintaining the health and integrity of the plants and associated animal communities in those habitats that remain. Established techniques toward this end are prescribed burns implemented by state and US Federal agencies. In contrast to the historical and natural burning that occurred every one to ten years during the growing season [77], these regular, controlled burns are often conducted in the winter (i.e., non-growing season). While it is assumed that the endemic members of this community, such as *E. semicrocea*, are most likely fire adapted [78], it remains unclear how these unnatural winter burns may impact the overall composition of pitcher plant bog communities. However, detection of high mortality among larvae with even a low intensity, patchy fire and further observations of *E. semicrocea* overwintering only as 4^th^ or 5^th^ instar larva [78] suggest winter burns are potentially detrimental to local populations. For example, *E. semicrocea* was not found at four sites (i.e., Centre, AL; Prattville, AL; Reed Branch, GA and Eller Seep, NC) visited for this study in spite of previously being reported as occurring at these locations (pers. comm. D. Folkerts and M. Hodges). We propose that previous winter burns conducted at these four sites in conjunction with isolation and fragmentation may have contributed to the potential extirpation of their *E. semicrocea* populations. This is alarming as there are other endemic pitcher plant arthropods that are considerably less common, and with potentially weaker dispersal capabilities, that may also be adversely impacted by such management techniques. This highlights the need for additional research specifically addressing the effects of fire on other constituents of pitcher plant bog communities and in the interim and until more information is gathered, it may be prudent to maintain unburned habitat during prescribed fire events (preferably during the growing season) as refugia for community members susceptible to such management practices.

Although pitcher plants have received attention with several species protected and others listed as endangered, threatened or of special concern, their arthropod inhabitants have received little or no conservation consideration. Because many of these arthropods are obligate associates of pitcher plants, they have likely suffered equivalent declines. Despite the fact that none have official protection status and the roles and importance that these endemic arthropods play within these habitats are poorly understood, conservation efforts should ideally strive to protect all components of threatened ecosystems. Due to the obvious effects that *E. semicrocea* has on *Sarracenia* spp. (i.e., leaf herbivory), this species has been one of the more studied [24], [25], [27]. Thus, the identification here of *E. semicrocea* as encompassing six distinct population groups within two divergent lineages (i.e., western and eastern) is of importance and should be considered when developing management and conservation strategies for pitcher plant bogs in the southeastern United States Coastal Plain. In this context, the three populations of *E. semicrocea* west of the Mississippi River (KN, AN, BT) are distinctive due to their strong genetic structure and this is further supported by the comparative phylogeographies of *E. semicrocea*, *S. alata*, and the microbial community within the pitchers representing unique and coevolving units in the WGCP (see above). Thus, steps should be taken to maintain the distinct genetic diversity at each of these sites. Similarly, the three population groups (i.e., EGCP, CF, SACP) of *E. semicrocea* in the eastern portion of its range deserve comparable attention. Overall, the two *E. semicrocea* lineages (i.e., west and east) could minimally be considered Evolutionary Significant Units (ESUs) of the species. The recognition of ESUs, defined as one or a set of populations with a distinct evolutionary heritage, can aid managers in prioritizing sites and activities related to conservation [79]. This region of the southeastern Coastal Plain has the highest biodiversity in the United States [80] and therefore future protection and/or restoration of this area has further implications for not only pitcher plant bogs and their constituents, but also other taxa [81], [82] and unique habitats [83] as well.

## Supporting Information

Table S1 **Distributions of** ***Exyra semicrocea*** **haplotypes by sampling localities/populations across the southeastern United States Coastal Plain.**
(DOC)
