# Supplementary material for: Genetic Differentiation, Structure, and a Transition Zone among Populations of the Pitcher Plant Moth Exyra semicrocea: Implications for Conservation
Source: PLoS One. 2011 Jul 28;6(7):e22658. doi: 10.1371/journal.pone.0022658 (PMC3145663; doi:10.1371/journal.pone.0022658)
Supplement: Table S1 — Distributions of Exyra semicrocea haplotypes by sampling localities/populations across the southeastern United States Coastal Plain. (DOC) [file pone.0022658.s001.doc]

|  |  | Sampling Localities/Populations | | | | | | | | | | |  |
| --- | --- | --- | --- | --- | --- | --- | --- | --- | --- | --- | --- | --- | --- |
| Haplotype Name | GenBank Accession | BT | AN | KN | AC | GB | WB | CF | AP | OF | FM | GS | Total # individuals/ haplotype |
| AC10 | HQ646110 |  |  |  | 1 |  |  |  |  |  |  |  | 1 |
| AC14 | HQ646111 |  |  |  | 1 |  |  |  |  |  |  |  | 1 |
| AC16 | HQ646112 |  |  |  | 1 |  |  |  |  |  |  |  | 1 |
| AC18 | HQ646113 |  |  |  | 4 |  | 1 |  |  |  |  |  | 5 |
| AC21 | HQ646114 |  |  |  | 1 | 1 |  |  |  |  |  |  | 2 |
| AC24 | HQ646115 |  |  |  | 2 |  |  | 2 |  |  |  |  | 4 |
| AC26 | HQ646116 |  |  |  | 1 |  |  |  |  |  |  |  | 1 |
| AC28 | HQ646117 |  |  |  | 1 |  |  |  |  |  |  |  | 1 |
| AC3 | HQ646118 |  |  |  | 1 |  |  |  |  |  |  |  | 1 |
| AC30 | HQ646119 |  |  |  | 1 |  |  |  |  |  |  |  | 1 |
| AC7 | HQ646120 |  |  |  | 7 | 6 | 16 | 3 | 1 | 3 |  |  | 36 |
| AC8 | HQ646121 |  |  |  | 1 | 3 |  | 2 |  |  |  |  | 6 |
| AN1 | HQ646122 |  | 2 |  |  |  |  |  |  |  |  |  | 2 |
| AP3 | HQ646123 |  |  |  |  |  | 1 |  | 2 | 12 | 7 | 4 | 26 |
| APA | HQ646124 |  |  |  |  |  |  |  | 1 |  |  |  | 1 |
| APC1 | HQ646125 |  |  |  |  |  |  |  | 1 |  |  |  | 1 |
| APF2 | HQ646126 |  |  |  |  |  |  |  | 1 |  |  |  | 1 |
| API3 | HQ646127 |  |  |  |  |  |  |  | 1 |  |  |  | 1 |
| APN | HQ646128 |  |  |  |  |  |  |  | 1 |  |  |  | 1 |
| APO | HQ646129 |  |  |  |  |  |  |  | 1 |  |  |  | 1 |
| APP1 | HQ646130 |  |  |  |  |  |  |  | 1 |  |  |  | 1 |
| APR1 | HQ646131 |  |  |  |  |  |  |  | 1 |  |  |  | 1 |
| BT11 | HQ646132 | 2 |  |  |  |  |  |  |  |  |  |  | 2 |
| BT14 | HQ646133 | 2 |  |  |  |  |  |  |  |  |  |  | 2 |
| BT19 | HQ646134 | 1 |  |  |  |  |  |  |  |  |  |  | 1 |
| BT22 | HQ646135 | 12 |  |  |  |  |  |  |  |  |  |  | 12 |
| BT3 | HQ646136 | 7 |  |  |  |  |  |  |  |  |  |  | 7 |
| CFF1 | HQ646137 |  |  |  |  | 1 |  | 5 | 1 |  |  |  | 7 |
| CFF6 | HQ646138 |  |  |  | 1 |  |  | 5 | 7 | 4 | 4 | 10 | 31 |
| CFL28 | HQ646139 |  |  |  |  | 3 | 2 | 2 | 2 |  |  |  | 9 |
| CFL37 | HQ646140 |  |  |  |  |  |  | 3 | 1 |  |  |  | 4 |
| CFL9 | HQ646141 |  |  |  |  |  |  | 2 |  |  |  |  | 2 |
| FM10 | HQ646142 |  |  |  |  |  |  |  |  |  | 3 |  | 3 |
| FM4 | HQ646143 |  |  |  |  |  |  |  |  |  | 1 |  | 1 |
| GBA2 | HQ646144 |  |  |  |  | 1 |  |  |  |  |  |  | 1 |
| GBC | HQ646145 |  |  |  |  | 1 |  |  |  |  |  |  | 1 |
| GBF | HQ646146 |  |  |  |  | 1 |  |  |  |  |  |  | 1 |
| GBI | HQ646147 |  |  |  |  | 1 |  |  |  |  |  |  | 1 |
| GBJ1 | HQ646148 |  |  |  |  | 1 |  |  |  |  |  |  | 1 |
| GBN | HQ646149 |  |  |  |  | 1 |  |  |  |  |  |  | 1 |
| GBO1 | HQ646150 |  |  |  |  | 1 |  |  |  |  |  |  | 1 |
| GBO2 | HQ646151 |  |  |  |  | 1 |  |  |  |  |  |  | 1 |
| GS2 | HQ646152 |  |  |  |  |  |  |  |  |  |  | 1 | 1 |
| KNA1 | HQ646153 |  |  | 1 |  |  |  |  |  |  |  |  | 1 |
| KNA2 | HQ646154 |  |  | 4 |  |  |  |  |  |  |  |  | 4 |
| KND | HQ646155 |  |  | 5 |  |  |  |  |  |  |  |  | 5 |
| KNE | HQ646156 |  | 1 | 12 |  |  |  |  |  |  |  |  | 13 |
| KNG | HQ646157 |  |  | 1 |  |  |  |  |  |  |  |  | 1 |
| KNQ | HQ646158 |  |  | 1 |  |  |  |  |  |  |  |  | 1 |
| OF17 | HQ646159 |  |  |  |  |  |  |  |  | 1 |  |  | 1 |
| OF19 | HQ646160 |  |  |  |  |  |  |  | 2 | 1 |  |  | 3 |
| WB3 | HQ646161 |  |  |  |  |  | 2 |  |  |  |  |  | 2 |
|  |  | Sampling Localities/Populations | | | | | | | | | | |  |
| Haplotype Name | GenBank Accession | BT | AN | KN | AC | GB | WB | CF | AP | OF | FM | GS | Total # individuals/ haplotype |
| WB4 | HQ646162 |  |  |  | 1 | 1 | 1 |  |  |  |  |  | 3 |
| WB5 | HQ646163 |  |  |  |  |  | 1 |  |  |  |  |  | 1 |
| Individuals/locations | | 24 | 3 | 24 | 24 | 23 | 24 | 24 | 24 | 21 | 15 | 15 |  |
